# SENSITIVE TO FREEZING2 is crucial for growth of *Marchantia polymorpha* under acidic conditions

**DOI:** 10.1007/s10265-024-01564-x

**Published:** 2024-08-04

**Authors:** Shinsuke Shimizu, Koichi Hori, Kimitsune Ishizaki, Hiroyuki Ohta, Mie Shimojima

**Affiliations:** 1 School of Life Science and Technology, Tokyo Institute of Technology, 4259-B-65, Nagatsuta-cho, Midori-ku, Yokohama, 226-8501 Kanagawa Japan; 2 Graduate School of Science, Kobe University, Kobe, Japan

**Keywords:** Acidic stress, GGGT, *Marchantia polymorpha*, Oligogalactolipid, SFR2, Trigalactosyldiacylglycerol

## Abstract

Land plants have evolved many systems to adapt to a wide range of environmental stresses. In seed plants, oligogalactolipid synthesis is involved in tolerance to freezing and dehydration, but it has not been analyzed in non-vascular plants. Here we analyzed trigalactosyldiacylglycerol (TGDG) synthesis in *Marchantia polymorpha*. TGDG is synthesized by galactolipid: galactolipid galactosyltransferase [GGGT; SENSITIVE TO FREEZING2 (SFR2) in Arabidopsis]. We analyzed the subcellular localization and GGGT activity of two *M. polymorpha* SFR2 homologs (MpGGGT1 and MpGGGT2, each as a GFP-fusion protein) using a transient expression system in *Nicotiana benthamiana* leaves and found that MpGGGT1-GFP localized in the chloroplast envelope membrane. We produced mutants Mp*gggt1* and Mp*gggt2* and found that TGDG did not accumulate in Mp*gggt1* upon treatment of the thallus with acetic acid. Moreover, growth of Mp*gggt1* mutants was impaired by acetic acid treatment. Microscopy revealed that the acetic acid treatment of *M. polymorpha* plants damaged intracellular membranes. The fact that the effect was similar for wild-type and Mp*gggt1* plants suggested that MpGGGT has a role in recovery from damage. These results indicate that MpGGGT plays a crucial role in *M. polymorpha* growth under conditions of acid stress, which may have been encountered during the ancient terrestrial colonization of plants.

**Supplementary Information:**

The online version contains supplementary material available at 10.1007/s10265-024-01564-x.

## Introduction

Lipid remodeling of membranes is one of the mechanisms plants have evolved to cope with stresses. Among such stresses, the effect of low temperature on the composition of membrane glycerolipids and their fatty-acid side chains has been extensively analyzed in cyanobacteria and plants. Under cold stress, the proportion of unsaturated fatty-acid side chains in membrane glycerolipids increases whereas that of saturated fatty acids decreases (Hugly and Somerville 1992; Miquel et al. 1993). Low temperature also alters the relative abundance of specific membrane glycerolipids, which correlates with low-temperature tolerance. In Arabidopsis, SENSITIVE TO FREEZING2 (SFR2) was discovered by screening freezing-sensitive mutants (Thorlby et al. 2004). SFR2 is essential for freezing tolerance and localizes on the outer envelope membrane of chloroplasts (Fourrier et al. 2008; Heemskerk et al. 1986; Roston et al. 2014). SFR2 was later identified as a galactolipid: galactolipid galactosyltransferase (GGGT) (Moellering et al. 2010), which synthesizes digalactosyldiacylglycerol and oligogalactolipids such as trigalactosyldiacylglycerol (TGDG) and tetragalactosyldiacylglycerol from monogalactosyldiacylglycerols (Heemskerk et al. 1983). The processive activity of SFR2 results in a reduction in the amount of non-bilayer-forming monogalactosyldiacylglycerols and increases the amount of oligogalactolipids in membranes. In Arabidopsis, TGDG is produced during protoplast isolation, under freezing stress, under severe heat stress, and by mechanical wounding (Barnes et al. 2019; Mueller et al. 2017; Vu et al. 2014, 2015). TGDG also accumulates under many other conditions and in numerous plant species—for example, in ozone-fumigated spinach leaves (Sakaki et al. 1990), the resurrection plant *Boea hygroscopica* under dehydration stress (Navari-Izzo et al. 1994; Sgherri et al. 1994), the desiccation-tolerant plant *Craterostigma plantagineum* during desiccation (Gasulla et al. 2013), and tomato (*Solanum lycopersicum*) under drought or salinity stress (Wang et al. 2016). In Arabidopsis, however, the activation of TGDG synthesis is not induced upon upregulation of *SFR2*. Subsequent work revealed that cytosolic acidification is the trigger for the activation of SFR2 function, which ultimately results in TGDG accumulation (Barnes et al. 2016). Moreover, to explore the evolution of the response to freezing stress, Barnes et al. (2023) investigated oligogalactolipid production in species ranging from bryophytes to angiosperms using TGDG production as a marker, revealing that cytosolic acidification is a common signal for TGDG production among multiple angiosperm species. Cytosolic acidification of plant cells can be induced by direct treatment with acid, but the extent of acidification and downstream effects vary depending on the type of acid and application method. TGDG accumulation was observed in detached leaves of Arabidopsis or pea after treatment with organic acids including acetic acid but not with inorganic acids (Barnes et al. 2016). The growth of Arabidopsis plants on low-pH medium adjusted with HCl or H_2_SO_4_ resulted in reduced fresh weight of seedlings and increased digalactosyldiacylglycerol content in chloroplast membranes or extraplastidic membranes compared with plants grown under control conditions, yet TGDG did not accumulate in the membranes of plants grown under either condition (Murakawa et al. 2019).

SFR2 is involved in more processes than just TGDG synthesis; in response to low temperature, for example, triacylglycerol (TAG) also accumulates in vegetative tissues. SFR2 supplies diacylglycerol (DAG) as a substrate for TAG synthesis mediated by multiple TAG synthases (Arisz et al. 2018; Barnes et al. 2016; Moellering et al. 2010; Roston et al. 2014; Shomo et al. 2024).

In our present study, we explored the evolution of stress-tolerance mechanisms mediated by SFR2, focusing on TGDG synthesis in the liverwort *Marchantia polymorpha* L. We first identified a *M. polymorpha* gene encoding GGGT among two homologous proteins of Arabidopsis SFR2 and then produced three lines of knockout plants by genome editing. Treatment of wild-type (WT) *M. polymorpha* plants with acetic acid induced TGDG accumulation in thalli, which was not observed in knockouts of *M. polymorpha* GGGT (Mp*gggt*). Moreover, in the Mp*gggt* knockout plants, treatment of plants with acetic acid resulted in a relatively severe phenotype, i.e., chlorosis of thalli. Comparison of growth between WT and Mp*gggt* knockout plants after plants were treated with acetic acid indicated that GGGT is crucial for *M. polymorpha* growth under acid stress.

## Materials and methods

### Plant materials and growth conditions

*Marchantia polymorpha* subsp. *ruderalis* Bischl. & Boisselier (henceforth denoted as Marchantia) plants of Takaragaike-1 accession (https://www.ncbi.nlm.nih.gov/nucleotide/AP031343.1, Tak-1; Ishizaki et al. 2008) were kindly provided by Dr. Takayuki Kohchi in Kyoto University and used as the wild type in this study. Thalli were maintained asexually with half-strength Gamborg’s B5 medium containing 1% (w/v) agar (hereafter, control medium). For sucrose medium, control medium was supplemented with 1% sucrose. For acetic acid medium, control medium was supplemented with 20 mM acetic acid and 10 mM MgCl_2_, and the pH was adjusted to pH 4.0 with KOH. Cultivation conditions were 23 °C with continuous light. All experiments were performed in accordance with relevant guidelines and regulations.

### Bioinformatics analyses

The Arabidopsis SFR2 sequence (TAIR10: AT3G06510.2) was used as a BLASTP query against multiple protein databases in Phytozome v13 (Goodstein et al. 2012), PhycoCosm (Grigoriev et al. 2021), and MarpolBase (Bowman et al. 2017) with an e-value cut-off of 1e^–50^ for identifying SFR2-like proteins. After removing redundant sequences and sequences with large deletions, the remaining sequences were used for protein domain analysis and phylogenetic analysis (Table S1). Protein domain analysis was performed using InterPro (Paysan-Lafosse et al. 2023). The amino-acid sequences were aligned using the MAFFT L-INS-i method (Katoh and Standley 2013) and subsequently trimmed with the trimAL v1.2 heuristic method (Capella-Gutiérrez et al. 2009) for phylogenetic analysis. The best substitution model was estimated with Aminosan (Tanabe 2011), and the Maximum likelihood tree was constructed using MEGA11 (Tamura et al. 2021).

### Transient expression in *Nicotiana Benthamiana*

Using cDNA prepared from Marchantia as the template, sequences encoding MpGGGT1 (Mapoly0094s0053, NLM-NCBI accession number PTQ32879) and MpGGGT2 (Mapoly0082s0065, NLM-NCBI accession number PTQ34235) (to which the attB sequence was added) were amplified (Table S2) by PCR and purified and subcloned into pDONR/Zeo (Invitrogen) using the BP reaction. The accuracy of the subcloned sequence was confirmed via DNA sequence analysis, and the subcloned sequence was inserted into pGWB5 using the LR reaction. Agrobacterium-mediated transient expression of genes was carried out as described by Wydro et al. (2006).

### Generation of transgenic plants to isolate mutants

For CRISPR/Cas9 genome editing, each target locus of Mp*GGGT1* and Mp*GGGT2* was selected with Casfinder (Aach et al. 2014) and CasOT (Xiao et al. 2014). The guide RNA sequences (Table S3) were annealed and ligated into pMpGE_En03 that had been digested with *Bsa* I (New England Biolabs, MA, USA). Using Gateway LR Clonase II (Thermo Fisher Scientific, USA) the guide RNA expression cassette was transferred into pMpGE010. Transformation of Marchantia was mediated by *Agrobacterium tumefaciens* GV3101 (pMP90) carrying a genome-editing vector and was performed according to Kubota et al. (2013).

### Treatment of Marchantia plants with acetic acid

For transient treatment, thalli were cultivated for 2 weeks in sucrose medium and soaked in acetic-acid solution (20 mM acetic acid/10 mM MgCl_2_, adjusted to pH 4.0 with K_2_HPO_4_; Barnes et al. 2016) for 1–6 h. After acetic acid treatment, thalli were collected and used for each experiment. For continuous growth observation after acetic acid treatment, thalli were grown transferred to the control medium.

### Lipid extraction

Total lipid was extracted from thalli or plant leaves according to Bligh and Dyer (1959) with the following modifications (Shimojo et al. 2023). Briefly, for whole-tissue samples, a maximum of 0.5 g fresh weight was frozen in liquid nitrogen. For the first extraction step, frozen thalli were crushed in liquid nitrogen and suspended with 3 mL chloroform: methanol (1:2 v/v). The suspension was centrifuged at 3,000 *g* for 5 min and the supernatant collected. The pellet after centrifugation was resuspended with 1.5 mL chloroform: methanol (1:2 v/v) and centrifuged at 3,000 *g* for 5 min and the supernatant collected. All supernatants (~ 4.5 mL each) were thoroughly mixed with 1.5 mL chloroform and 3 mL of 1% (w/v) KCl. Each mixture was centrifuged at 3,000 *g* for 5 min, and the organic phase (lower layer, containing the extracted total lipid) was collected and dried under N_2_ gas at 30 °C, and then the residue was dissolved in chloroform: methanol (2:1 v/v) and stored at − 20 °C.

### Lipid analysis

For detection of oligogalactolipids, polar glycerolipids were separated from 600 µg total lipid extract on a silica gel plate (105721, Merck KGaA, Darmstadt, Germany) by one-dimensional thin-layer chromatography with chloroform/methanol/acetic acid/water (85:20:10:4, v/v). After separation, the plate was sprayed with anthrone-sulfuric acid solution (0.05 g anthrone, 1 g thiourea in 66% (v/v) aqueous sulfuric acid) and heated at 110 °C for 15 min to visualize the polar glycerolipids.

For quantification of TAGs, TAGs were separated from 600 µg total lipid extract on a silica gel plate by one-dimensional thin-layer chromatography with hexane/diethyl ether/acetic acid (80:20:2, v/v). After separation, the plate was sprayed with 0.01% (w/v) primuline in 80% (v/v) acetone, and lipids were visualized under ultraviolet light. The silica containing TAGs was scraped from the plate and incubated with 600 µL of 5% (v/v) HCl methanolic solution (mixture of 300 µL methanol and 300 µL of 10% HCl solution; 90964, Sigma-Aldrich, USA) at 85 °C for 1 h with 100 µL of 1 mM heneicosanoic acid (H5149, Merck) as internal standard. Fatty-acid methyl esters were extracted with hexane, and the resulting dry fatty-acid methyl ester residue was suspended with 100 µL hexane. Fatty-acid methyl esters were separated on a capillary column (ULBON HR-SS-10, length, 25 m; internal diameter, 0.25 mm; Shinwa Chemical Industries Ltd., Kyoto, Japan) and detected by gas chromatography (model GC-2030, Shimadzu Co., Kyoto, Japan) equipped with a flame ionization detector. Nitrogen was used as both the carrier and make-up gas. The injection volume was 1 µL with a split ratio of 1:50. The injection port and detector temperatures were 250 °C. The linear velocity of the carrier gas was 13 cm s^–1^. The column temperature program was as follows: temperature was held at 180 °C for 26 min, increased to 184 °C at 1 °C min^–1^, increased to 188 °C at 5 °C min^–1^, increased to 195 °C at 1 °C min^–1^, increased to 200 °C at 5 min^–1^, increased to 210 °C at 3 °C min^–1^, and held at 210 °C for 2 min. Absolute amounts of fatty-acid methyl esters were calculated using the internal standard C21:0. Standard solutions for calibration curves were produced with fatty-acid methyl ester mixtures (Supelco 37 FAME mix, Nu-Chek Prep, Inc., MN, USA).

### Electron microscopy

Thalli segments were fixed with 2% paraformaldehyde and 2.5% glutaraldehyde in 0.067 M sodium phosphate (pH 7.4) for 2 h at room temperature and then for 16 h at 4 °C. Samples were washed six times with the same buffer for 10 min each time at room temperature. Samples were then post-fixed with 2% osmium tetroxide in 0.067 M sodium phosphate (pH 7.4) for 2 h at room temperature. The fixed samples were dehydrated in a graded ethanol series and embedded in epoxy resin mixture (Quetol 651 mixture, Nissin EM, Tokyo). Ultrathin sections were cut with a diamond knife on a Leica UC7 ultramicrotome and transferred onto copper grids. The sections were stained with Mayer’s hematoxylin for 10 min followed by lead citrate for 10 min at room temperature. The specimens were observed on a JEOL 1400plus transmission electron microscope at an accelerating voltage of 80 kV. The center part of *gggt1* mutant thalli was used for electron microscopy because the peripheral parts of the mutant thalli were chlorotic even on the first day after treatment with acetic acid.

## Results

### Conserved sequences and phylogenetic analyses of SFR2-like proteins

**Fig. 1:**
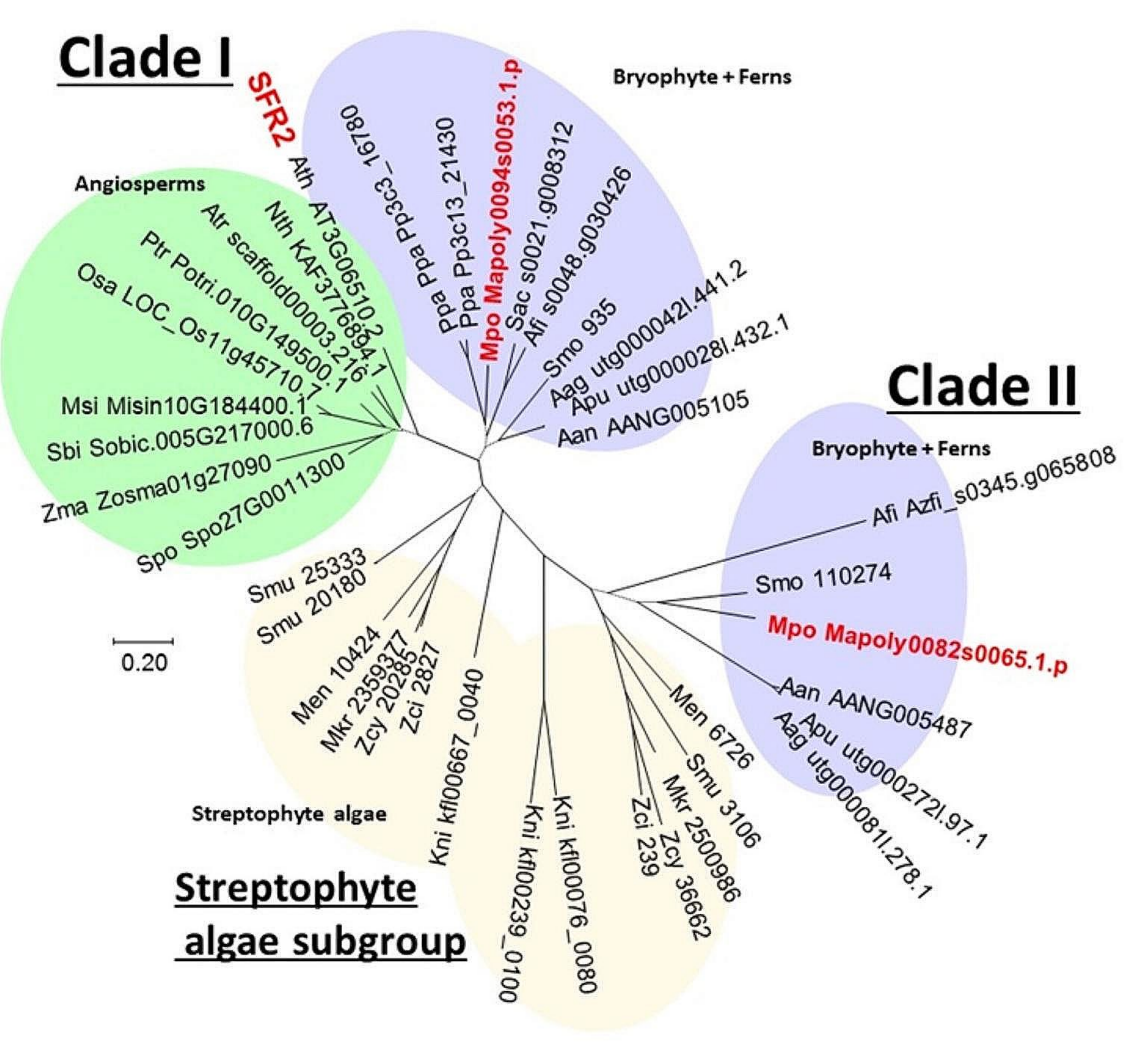
Maximum likelihood unrooted phylogenetic tree of SFR2-like proteins of streptophyte algae and land plants. The phylogenetic tree was constructed with MEGA11 using the maximum likelihood method based on the LG + G (8) model. The scale bar indicates the number of substitutions per site. Bootstrap analysis with 500 replicates was carried out to estimate the support for internal nodes. Dashed lines indicate internal branches with support values below 70%. Abbreviations for organisms and the protein IDs (Table S1) are displayed in leaf nodes. Organismal groups are color-shaded as follows: angiosperms, green; bryophytes and ferns, violet; streptophyte algae, yellow. Kni, *Klebsormidium nitens*; Men, *Mesotaenium endlicherianum*; Mk, *Mesotaenium kramstae Lemmermann*; Zcy, *Zygnema* cf. *cylindricum*; Zci, *Zygnema curcumcarinatum*; Smu, *Spriogolea muscicola*; Aag, *Anthoceros agrestis*; Aan, *Anthoceros angustus*; Apu, *Anthoceros punctanus*; Mpo, *Marchantia polymorpha*; Ppa, *Physcomitrium patens*; Smo, *Selaginella moellendorfii*; Afi, *Azolla fillculoides*; Scu, *Salvinia cucullate*; Atr, *Amborella trichopoda*; Nth, *Nymphaea thermarum*; Ath, *Arabidopsis thaliana*; Pt, *Populus trichocarpa*; Zma, *Zostera marina*; Msi, *Miscanthus sinesis*; Sbi, *Sorghum bicolor*; Osa, *Oryza sativa*; Spo, *Spirodela polyrhiza*

To identify *GGGT* genes of Marchantia, we examined conserved amino-acid sequences and carried out a phylogenetic analysis using Arabidopsis *SFR2* and 37 *SFR2*-like genes selected from 17 land plants and 6 streptophyte algae including *Klebsormidium nitens*, in which the presence of TGDG-like lipid in the membranes was suggested (Hori et al. 2016) (Table S1). The phylogenetic tree showed that SFR2-like proteins diverged in the streptophyte algae subgroup and can be divided into two major clades of land plants (Fig. 1). Among the selected species, Clade I comprised SFR2 and was found across all land-plant lineages. In contrast, Clade II comprised only bryophytes and ferns. All SFR2-like proteins were found to retain the glycosyl hydrase I domain (IPR001360), with conservation of the active-site glutamates and nearby hydrophobic patch required for GGGT activity (Roston et al. 2014). The Loop A region of SFR2 has also been reported to play a crucial role in GGGT activity, but this loop is predicted as an intrinsically disordered region (Roston et al. 2014) and exhibited significant diversity among different SFR2-like proteins. However, the Loop A regions of Clade I SFR2-like proteins in bryophytes and ferns tended to be longer than those of angiosperms, whereas the Loop A regions of Clade II SFR2-like proteins were significantly shorter (Fig. S1). Two *SFR2*-like genes, namely Mapoly0094s0053 and Mapoly0082s0065, belonging to Clade I and Clade II, respectively, were identified in the Marchantia genome, and it was expected that either or both of the encoded enzymes would exhibit GGGT activity. Hereafter, these two respective genes are denoted as Mp*GGGT1* and Mp*GGGT2*.

### Transient expression of MpGGGT1-GFP in *N. benthamiana* leaves results in its chloroplast localization, like SFR2-GFP

**Fig. 2:**
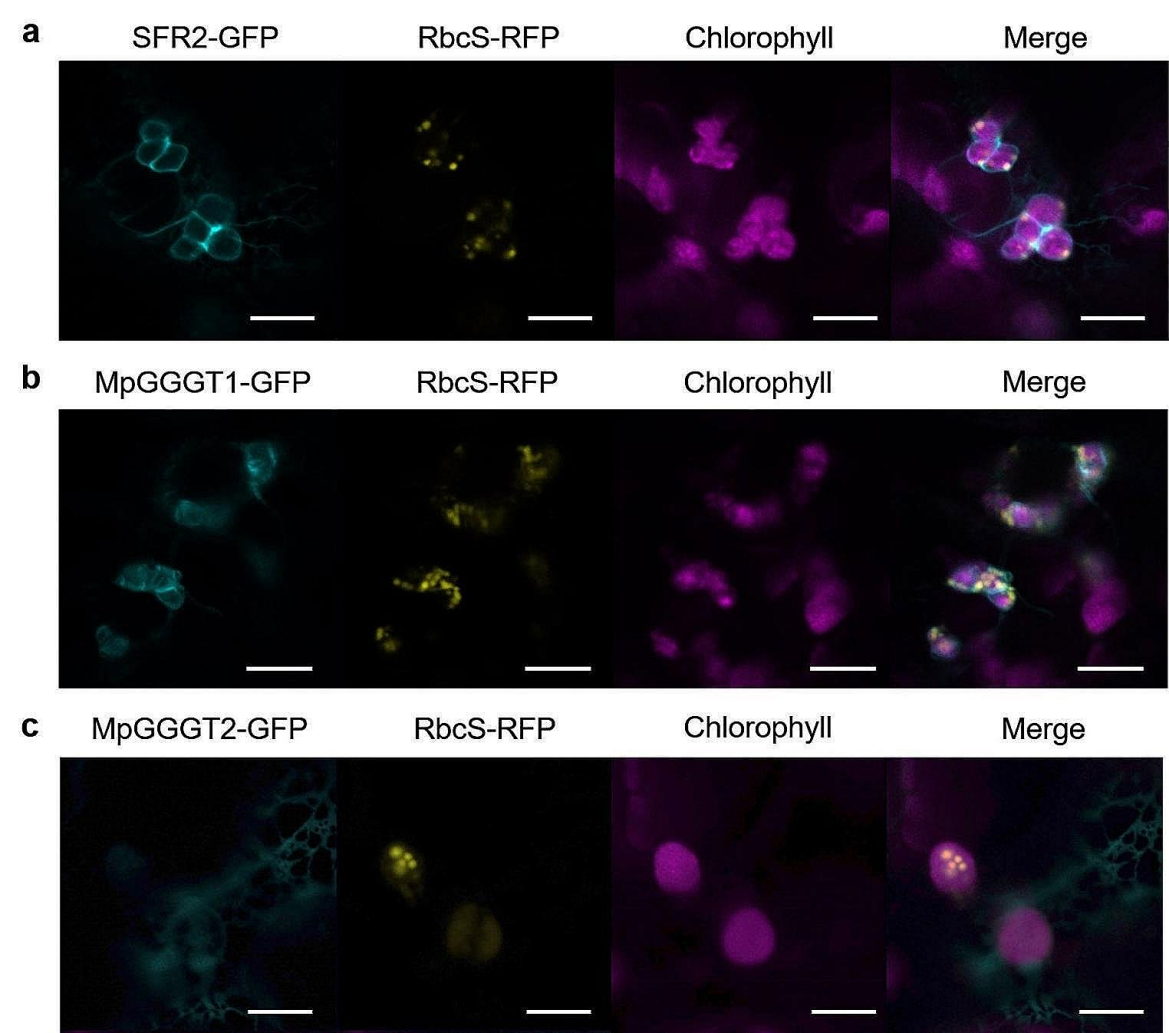
Transient expression of MpGGGTs in *N. benthamiana* leaves and their subcellular localization. Subcellular localization of SFR2-GFP (**a**), MpGGGT1-GFP (**b**) and MpGGGT2-GFP (**c**) was assessed by confocal laser microscopy. All MpGGGTs were coexpressed with RbcS-RFP (Rubisco small subunit, as a marker protein for chloroplast stroma localization). Cyan, GFP; yellow, red fluorescent protein (RFP); magenta, chlorophyll autofluorescence. Scale bars = 10 μm

MpGGGT1 and MpGGGT2 (each tagged with green fluorescent protein, GFP) were transiently expressed in *N. benthamiana* leaves, and subcellular localization was analyzed by confocal fluorescence microscopy. Co-expression of Arabidopsis rubisco small subunit (tagged with red fluorescent protein) was used to mark the stroma. As previously reported by Fourrier et al. (2008), SFR2-GFP localized in the chloroplast envelope membrane. MpGGGT1-GFP localization was similar to that of SFR2-GFP, suggesting that MpGGGT1-GFP localized in the chloroplast envelope membrane. In contrast, MpGGGT2-GFP showed weak localization in the cytoplasm (Fig. 2).

### Transient expression of MpGGGT1-GFP results in TGDG production in *N. benthamiana* leaves

**Fig. 3:**
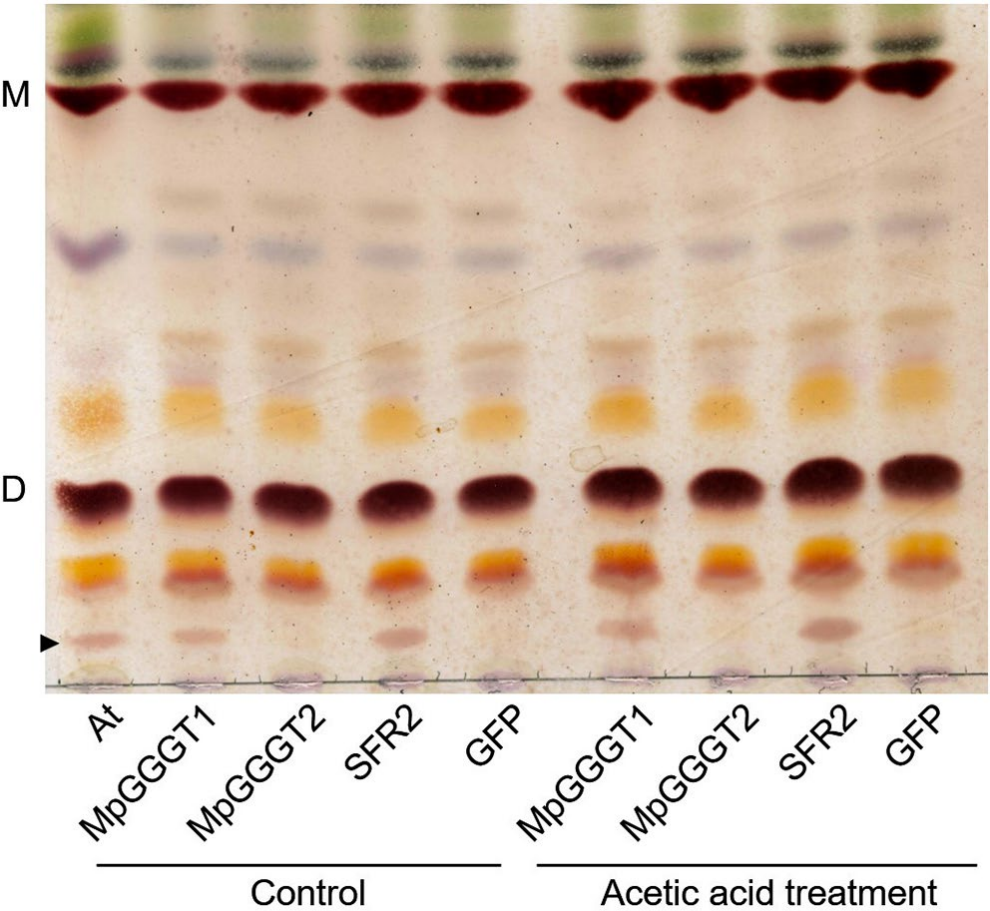
Lipid analysis of *N. benthamiana* leaves transiently expressing MpGGGT1-GFP or MpGGGT2-GFP. One-dimensional thin-layer chromatography analysis of 600 µg total lipids from acetate-treated *N. benthamiana* leaves expressing MpGGGT1-GFP, MpGGGT2-GFP, SFR2-GFP, or GFP. At, total lipid from *A. thaliana* leaves treated with acetic acid; MpGGGT1, MpGGGT1-GFP; MpGGGGT2, MpGGGT2-GFP; SFR2, SFR2-GFP. GFP; GFP control. Black arrowhead indicates TGDG. M, MGDG; D, DGDG

It has been reported that soaking Arabidopsis rosette leaves in acetic-acid solution results in TGDG accumulation and GGGT activity, dependent on Mg^2+^ concentration in vitro (Barnes et al. 2016). Thus, we assessed TGDG content in *N. benthamiana* leaves expressing MpGGGT1-GFP, MpGGGT2-GFP, SFR2-GFP, or GFP before and after treatment of leaves with acetic-acid solution containing Mg^2+^ (Fig. 3). Expression of GFP alone did not result in TGDG accumulation, even with acid treatment, suggesting that endogenous *N. benthamiana* GGGT was insufficient to accumulate TGDG. In leaves expressing MpGGGT1-GFP or SFR2-GFP, however, TGDG accumulated even in the absence of acid treatment (Fig. 3). Notably, TGDG did not accumulate in leaves expressing MpGGGT2-GFP, even with acid treatment. These results suggested that MpGGGT1—among all SFR2-like proteins of Marchantia—is involved in TGDG synthesis.

### Knockout of MpGGGT1 or MpGGGT2 does not affect Marchantia plant growth under control conditions

**Fig. 4:**
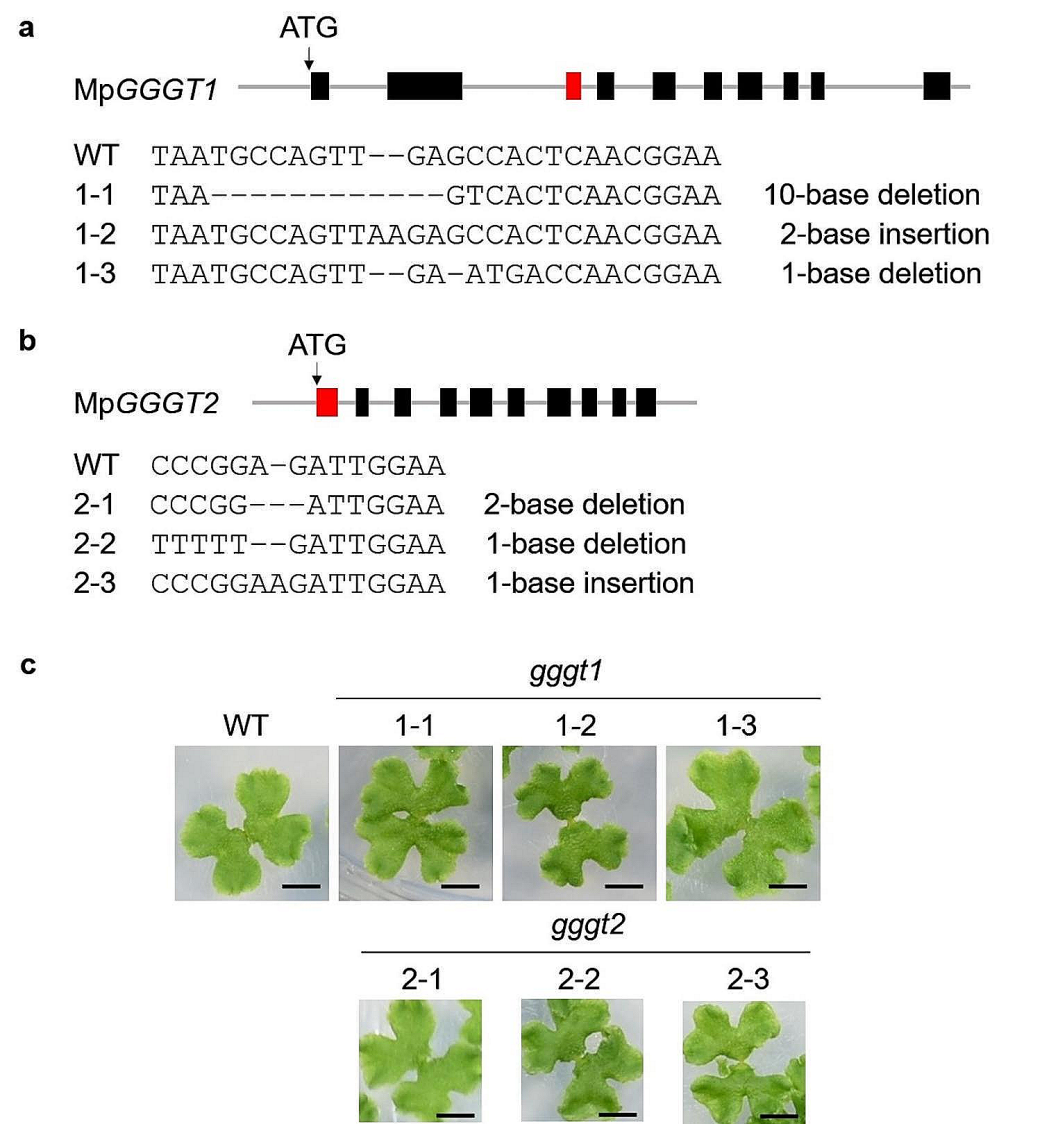
Genome editing of Mp*GGGT1* and Mp*GGGT2* by CRISPR-Cas9. **a** The third exon of Mp*GGGT1* (red box) was targeted for genome editing via the CRISPR-Cas9 system. The genome sequence is indicated for each of WT Mp*GGGT1* and Mp*GGGT1* mutants (Mp*gggt1*) 1–1, 1–2 and 1–3. **b** The first exon of Mp*GGGT2* (red box) was targeted for genome editing via the CRISPR-Cas9 system. The genome sequence is indicated for each of WT Mp*GGGT2* and Mp*GGGT2* mutants (Mp*gggt2*) 2 − 1, 2–2 and 2–3. Black boxes, exons; gray lines, introns. **c** Growth of WT, Mp*gggt1* and Mp*gggt2* mutant strains at 14 days. Scale bars = 0.5 cm

To further investigate GGGT activity and the physiological role of MpGGGT1 and MpGGGT2 in Marchantia, we generated three independent loss-of-function (knockout) mutants of each gene (Fig. 4). The CRISPR/Cas9 system was used to carry out genome editing of the third exon of Mp*GGGT1* and the first exon of Mp*GGGT2*, which yielded three knockout lines of each named *gggt1* (1–1, 1–2, and 1–3) for MpGGGT1 (Fig. 4a) and *gggt2* (2 − 1, 2–2, and 2–3) for MpGGGT2 (Fig. 4b). For each of the three Mp*gggt1* mutants, a frame-shift resulted in the conversion of a conserved amino acid in the active site (based on the SFR2 sequence; Roston et al. 2014) to a different amino acid. For each of the Mp*gggt2* mutants, the transcript contained a stop codon located just upstream of the predicted active site. Although these mutants were considered as loss-of-function mutants, the growth of each of the Mp*gggt1* and Mp*gggt2* mutant plants was similar to that of WT under control conditions (Fig. 4c).

### Acetic acid treatment enhances MpGGGT1-dependent TGDG accumulation in Marchantia

**Fig. 5:**
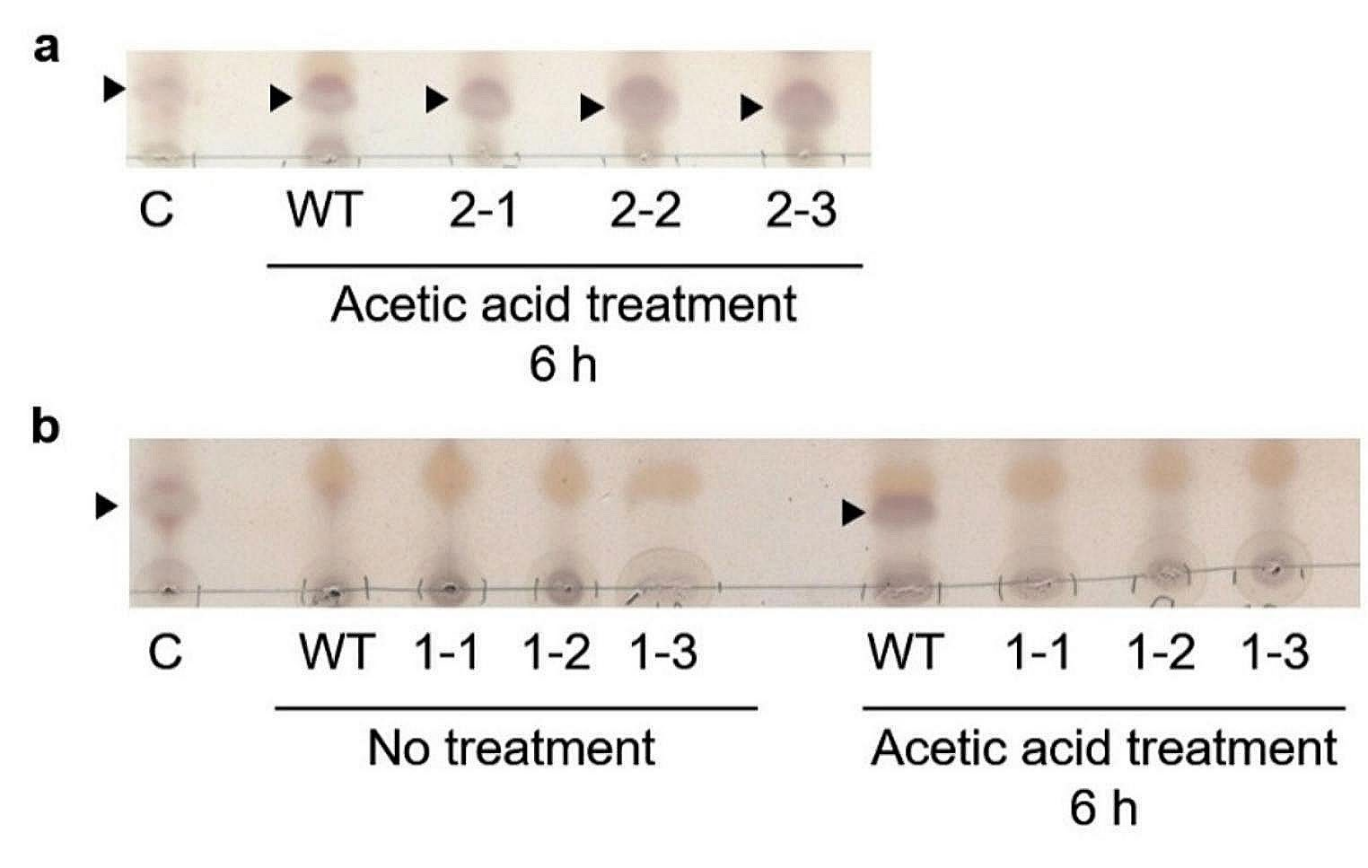
Lipid analysis of acetic acid-treated and non-treated *M. polymorpha* WT, Mp*gggt1*, and Mp*gggt2* mutants. One-dimensional thin-layer chromatography analysis of 600 µg total lipid in acetic acid–treated (6 h) and non-treated thalli of WT and Mp*gggt2* mutants (**a**) or Mp*gggt1* mutants (**b**). Black arrowheads indicate TGDG. C, Total lipid from *A. thaliana* leaves after a 3-h treatment with acetic acid

To investigate whether MpGGGT1 is involved in TGDG synthesis in Marchantia, we soaked 2-week-old WT, Mp*gggt1* and Mp*gggt2* mutants in acetic-acid solution for 6 h and analyzed TGDG accumulation (Fig. 5). In WT plants, the acid treatment resulted in TGDG accumulation (Fig. 5a, black arrowheads) that was similar to that previously reported for acetic acid–treated Arabidopsis and pea (Barnes et al. 2016). Similarly, TGDG accumulated in the three acid-treated Mp*gggt2* mutants (Fig. 5a). For the three Mp*gggt1* mutants, however, TGDG did not accumulate even with acid treatment (Fig. 5b). These results clearly indicated that MpGGGT1 is the major GGGT responsible for TGDG accumulation induced by acetic acid treatment in Marchantia.

### GGGT is required for growth of acetic acid–treated Marchantia plants

**Fig. 6:**
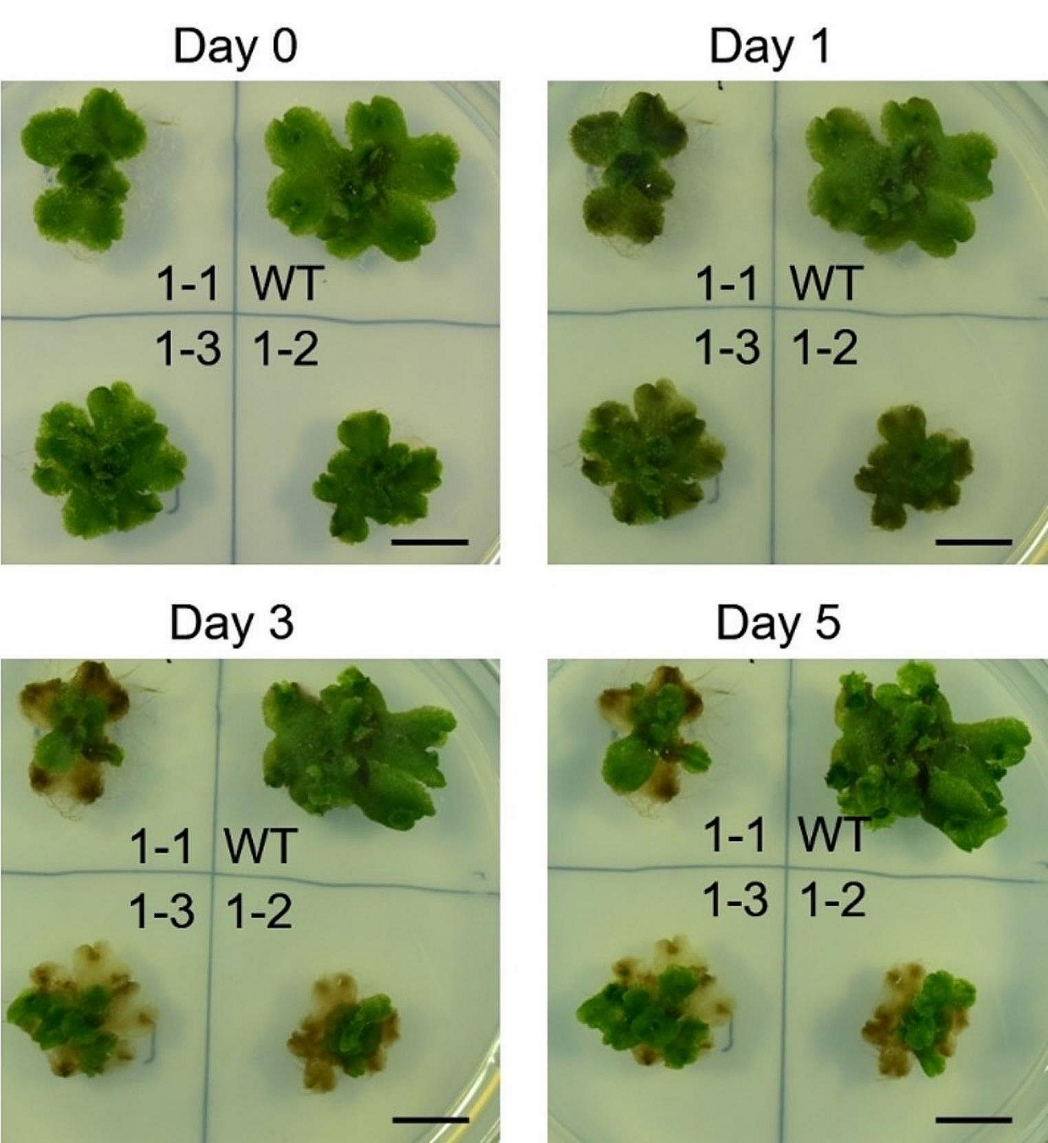
Growth phenotypes of *M. polymorpha* WT and Mp*gggt1* mutants after transfer from acetic acid medium to control medium. Growth of the *M. polymorpha* immediately after transfer from acetic acid medium to control medium (Day 0) or on Days 1, 3, and 5 after the transfer. Scale bars = 1 cm

Next, we compared the growth of WT and Mp*gggt1* mutant plants grown on sucrose medium for 2 weeks, transferred to acetate-containing medium for 6 h, and then transferred to control medium (Fig. 6). On the day of transfer to control medium (Fig. 6, Day 0), the WT and Mp*gggt1* mutant plants were morphologically similar. The next day (Fig. 6, Day 1), however, the area around the thallus of Mp*gggt1* mutants began to show chlorosis, which was not observed for WT. On Days 3 and 5, most of the Mp*gggt1* mutant thalli were chlorotic whereas WT thalli were unaffected. These results indicated that GGGT-mediated TGDG synthesis is crucial for Marchantia growth after treatment with acetic acid.

### GGGT is also involved in TAG accumulation in acetic acid–treated Marchantia plants

**Fig. 7:**
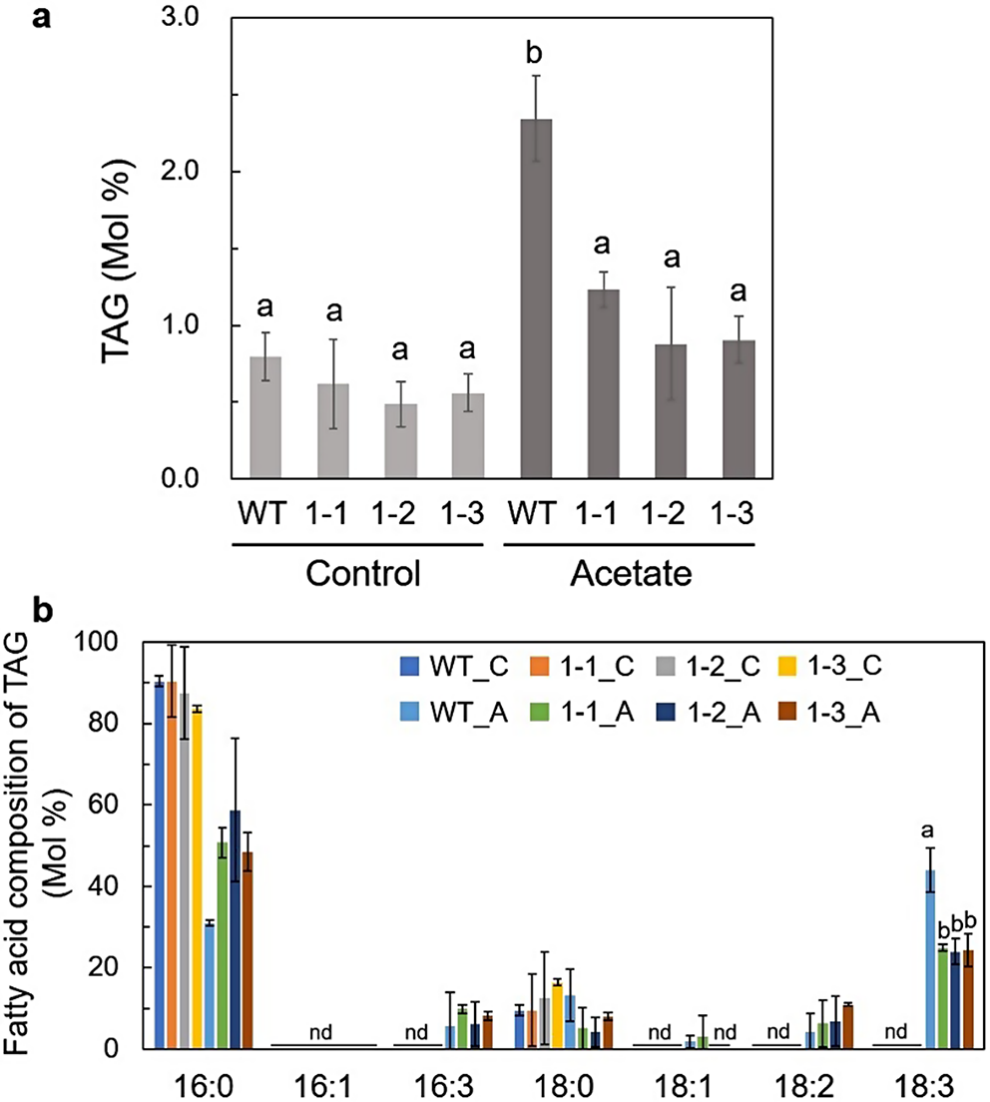
TAG content and the fatty-acid composition of acetate-treated and non-treated thalli of WT and Mp*gggt1* mutants. **a** Percentage of TAG-derived fatty acids among total fatty acids. Control, non-treated; Acetate, acetate-treated. **b** Percentage of each fatty-acid species in TAG as a percentage of the total fatty acids in TAG. C, Non-treated; A, acetate-treated; nd, not detected. Different lowercase letters (**a**, **b**) indicate a significant difference between values (*P* < 0.05; Tukey’s test, *n* = 3)

In Arabidopsis, SFR2/GGGT is also involved in TAG synthesis by supplying DAG as a substrate for DGAT1 after freezing stress (Arisz et al. 2018; Moellering et al. 2010). To determine whether GGGT-mediated TGDG synthesis correlated with TAG accumulation in Marchantia plants, we analyzed the TAG content and the fatty-acid composition in WT and Mp*gggt1* mutants before and after acetic acid treatment. The TAG content under control conditions was comparable between WT and Mp*gggt1* mutant plants (Fig. 7a, Control). Treatment of WT plants with acetic acid resulted in a significant increase in TAG content, but the increase was not as pronounced in acetic acid–treated Mp*gggt1* mutant plants (Fig. 7a). We then compared the fatty-acid composition of TAG between WT and Mp*gggt1* mutants with or without acetic acid treatment (Fig. 7b). The acid treatment of WT plants significantly affected the fatty-acid composition of TAG, as evidenced by the decreased mole percentage of C16:0 fatty acids and increased percentage of C18:3. However, these changes were not as pronounced in the Mp*gggt1* mutants. TAG biosynthesis can be mediated by membrane-lipid degradation, particularly of monogalactosyldiacylglycerols; as such, any increase in the content of highly unsaturated fatty acids like C18:3 in TAG would indicate a shift toward lipid degradation because desaturation occurs after assembly of glycerolipids. These data for fatty-acid composition suggested that TAG biosynthesis—as mediated by membrane-lipid breakdown—increases in plants treated with acetic acid. Moreover, given that the fatty-acid composition of TAG in Mp*gggt1* mutant plants was less affected by acetic acid treatment, it was clear that GGGT contributes substantially to TAG biosynthesis induced by acetic acid treatment but that another process is responsible for the observed change in fatty-acid composition.

### Chloroplast degradation observed in Mp*gggt1* mutant plants is similar to that in WT

**Fig. 8:**
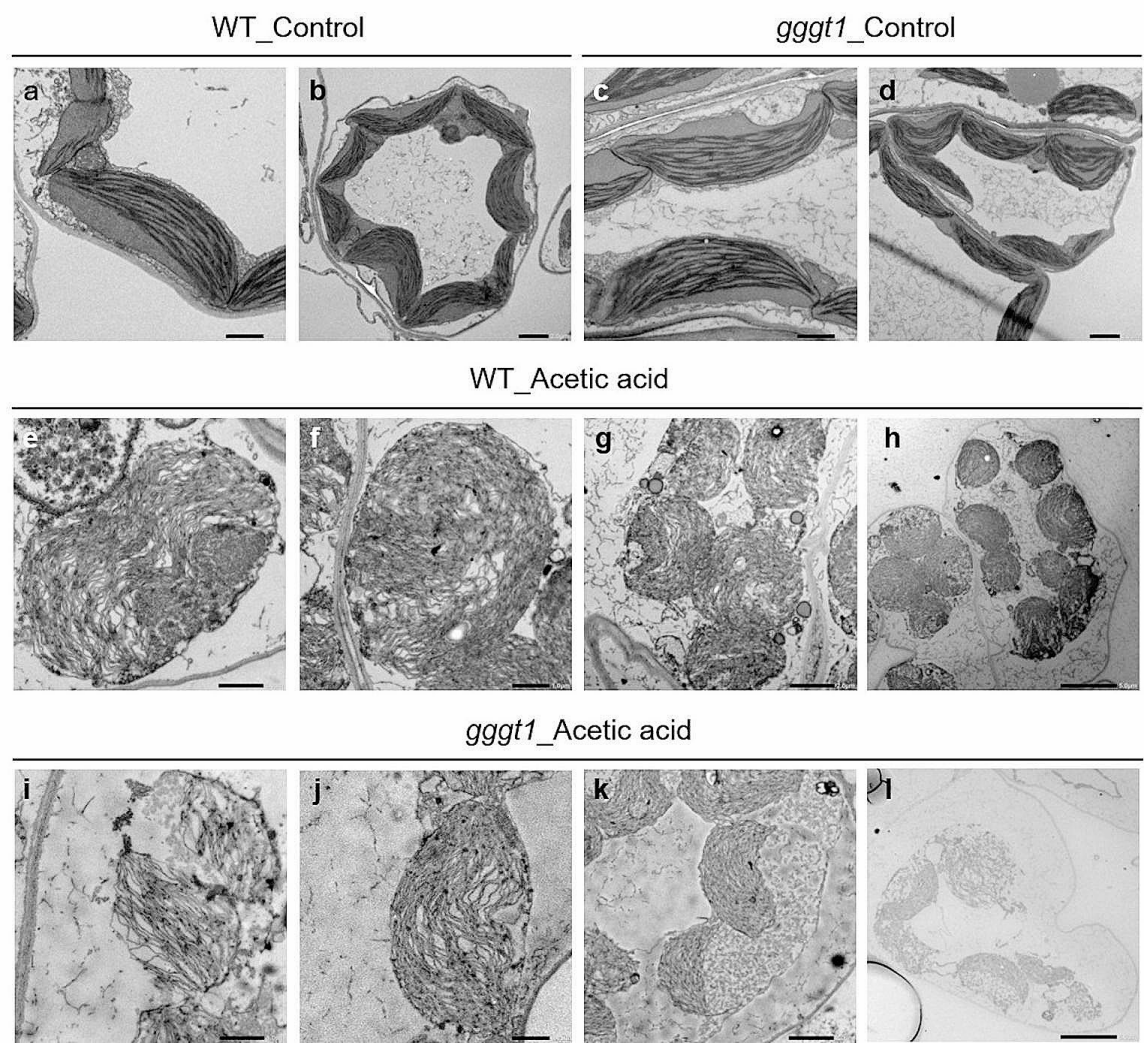
Intracellular membrane structure of WT and Mp*gggt1* mutant thalli observed by transmission electron microscopy. **a–d**: WT plants (**a**, **b**) and Mp*gggt1* 1–3 mutant plants (**c**, **d**) grown under control conditions. **e–l**: WT plants (**e–h**) and Mp*gggt1* mutant plants (**i** to **l**) were transferred from acetic acid medium to control medium. Scale bars (**a**, **c**, **e**, **f**, **i**, **j**) = 1 μm; (**b**, **d**, **g**, **k**) = 2 μm; (**h**, **l**) = 5 μm

SFR2-mediated TGDG synthesis followed by TAG synthesis changes the ratio of bilayer-to-non-bilayer-forming lipids in membranes (Moellering et al. 2010). Moellering and Benning also proposed a model that TGDG, with its large polar head, is synthesized in Arabidopsis to avoid membrane-membrane fusion (Moellering and Benning 2011). Thus, we used electron microscopy to compare the intracellular membrane structure of the Marchantia thallus between WT and Mp*gggt1* mutants before and after acetic acid treatment (Fig. 8). Under control growth conditions, WT and Mp*gggt1* mutants had similar intracellular membrane structures (Fig. 8a–d). After a 6-h treatment with acetic acid, however, both WT and Mp*gggt1* mutants had disrupted, swollen chloroplasts with concomitant disruption of vacuoles (Fig. 8e–l). The thylakoid membranes were also severely damaged. The fact that the degree of damage was similar between WT and Mp*gggt1* mutant cells suggested that the activation of GGGT followed by an increase in both TGDG and TAG content might contribute to chloroplast recovery after acetic acid treatment.

## Discussion

Our results show that TGDG accumulated in response to treatment of Marchantia thalli with acetic acid in the presence of Mg^2+^; however, the TGDG content was very low when the thallus was not treated with acetic acid. TGDG did not accumulate in Mp*gggt1* mutants even after acetic acid treatment, suggesting that TGDG accumulation resulted from GGGT activation in response to acid treatment. The accumulation of TGDG upon treatment with acetic acid and/or Mg^2+^ was initially described in Arabidopsis leaves (Barnes et al. 2016). In Arabidopsis, cytosolic pH decreases in response to freezing or treatment with acetic acid, and this cytosolic acidification activates SFR2 function; this response mechanism is conserved in species from bryophytes to angiosperms (Barnes et al. 2016, 2023). Given that Marchantia accumulated TGDG and TAG in response to acetic acid treatment in a GGGT-dependent manner (as was observed in Arabidopsis), it is possible that the severe-cold response mediated by GGGT is also conserved in Marchantia. As one example of acid stress, we treated Marchantia plants with acetic acid and assessed the growth of WT and Mp*gggt1* plants, revealing that the absence of GGGT function reduced tolerance to acetic acid and induced partial cell death of the thallus. In both WT and Mp*gggt1* mutants, treatment of Marchantia plants with acetic acid and Mg^2+^ caused severe damage to intracellular membrane structures—in particular, vacuolar and chloroplast membranes. The damage was similar in all cells of both WT and Mp*gggt1*. Barnes et al. (2016) suggested that both the leakage of Mg^2+^ from chloroplasts and increased availability of the substrate (MGDG) for SFR2 owing to disruption of the chloroplast membrane may constitute the mechanism for rapid activation of the membrane protective machinery mediated by SFR2 activation. However, in Marchantia thallus cells treated with acetic acid, the proposed membrane protection mediated by MpGGGT was not clearly observed by electron microscopy. Given that acetic acid had a greater negative impact on the growth of mutant plants than WT, it is possible that MpGGGT has a pivotal role in recovery from the damage caused by acetic acid treatment.

DAG is a byproduct of the processive reaction of GGGT, yet accumulation of DAG can disrupt membrane function and cause oxidative stress and even cell death, known collectively as lipotoxicity (Fan et al. 2013, 2017; Garbarino et al. 2009; Lu et al. 2020; Yu et al. 2021). To avoid the negative effects of DAG, an immediate conversion of DAG to TAG might occur in cells when SRF2 is activated in Arabidopsis. TAG also accumulated in Marchantia WT plants after treatment with acetic acid and Mg^2+^, but the accumulation was less pronounced in Mp*gggt1* plants. Analysis of the fatty-acid composition of TAG indicated that the TAG synthetic pathway, upon induction by acetic acid and Mg^2+^, differs from that under control conditions. Under control conditions, the abundance of polyunsaturated fatty acids such as 16:3, 18:2 and 18:3 in TAG was extremely low, and these species were not detected in WT or the mutant. After acetic acid treatment, however, the abundance of polyunsaturated fatty acids increased significantly in both the WT and the mutant plants, although the extent of the change in the mutants was lesser by approximately one-half. These results suggest that TAG biosynthesis—especially the origin of the substrate DAG—is altered after acetic acid treatment. We previously identified phosphatidic acid phosphohydrolase (PAH) in Marchantia and found that this PAH is involved in the same enzymatic reaction as Arabidopsis PAH, but the contribution of the Marchantia PAH to lipid metabolism appears to be limited to rhizoids, unlike that of Arabidopsis PAH (Shimojo et al. 2023). Based on these results, it might also be necessary to investigate the contribution of MpGGGT to lipid metabolism in rhizoids.

TGDG synthesis is widely conserved among land plants, and the SFR2-like GGGT is presumed to have evolved before the emergence of land plants (Barnes et al. 2016, 2023; Hori et al. 2016). TGDG accumulation under freezing, drought, or osmotic stress is beneficial for adaptation to dehydration under these stresses associated with terrestrial environments. Our results show that MpGGGT1 contributes to acetic acid tolerance in Marchantia. Organic acids, such as acetic acid, are widely distributed in plant secretions and humic substances, leading to the formation of acidic soils such as peat. Soil components having an appearance similar to that of peat moss have been discovered in microfossils from the Ordovician period (Cardona-Correa et al. 2016), during which the ancestors of mosses colonized land. The humic substances of soils, which contain an abundance of organic acids and have low pH, might have been disadvantageous during the expansion of land plants into new habitats. Thus, the ability to tolerate organic acids may have benefited the evolution of land plants with respect to their growth in soils harboring their own humic substances. In addition to the adaptation to dehydration stress by GGGT, an analysis of organic-acid tolerance of Marchantia species may yield useful information for future research.

## Electronic supplementary material

Below is the link to the electronic supplementary material.

Supplementary Material 1

## Data Availability

The datasets generated and/or analyzed during the current study are available in the DDBJ/EMBL/GenBank repository [https://www.ncbi.nlm.nih.gov/genbank/ for GenBank]. The accession numbers of DNA sequences from Mp*gggt1* 1–1, 1–2, 1–3, Mp*gggt2* 2 − 1, 2–2, and 2–3 are LC816730, LC816731, LC816732, LC816733, LC816734, and LC816735, respectively. The other data that support the findings of our study are available from the corresponding author upon reasonable request.
