# Supplementary material for: SENSITIVE TO FREEZING2 is crucial for growth of Marchantia polymorpha under acidic conditions
Source: J Plant Res. 2024 Aug 4;137(6):1115–26. doi: 10.1007/s10265-024-01564-x (PMC11525325; doi:10.1007/s10265-024-01564-x)
Supplement: Supplementary file 1 — Supplementary Material 1 [file 10265_2024_1564_MOESM1_ESM.pdf]

SENSITIVE TO FREEZING2 is crucial for growth of *Marchantia polymorpha* under acidic conditions

Journal of Plant Research

Shinsuke Shimizu[1], Koichi Hori[1], Kimitsune Ishizaki[2], Hiroyuki Ohta[1], Mie Shimojima[1,*]

[1]School of Life Science and Technology, Tokyo Institute of Technology, Yokohama, Japan, [2]Graduate School of Science, Kobe University, Kobe, Japan

*Corresponding author:
Mie Shimojima
shimojima.m.aa@m.titech.ac.jp

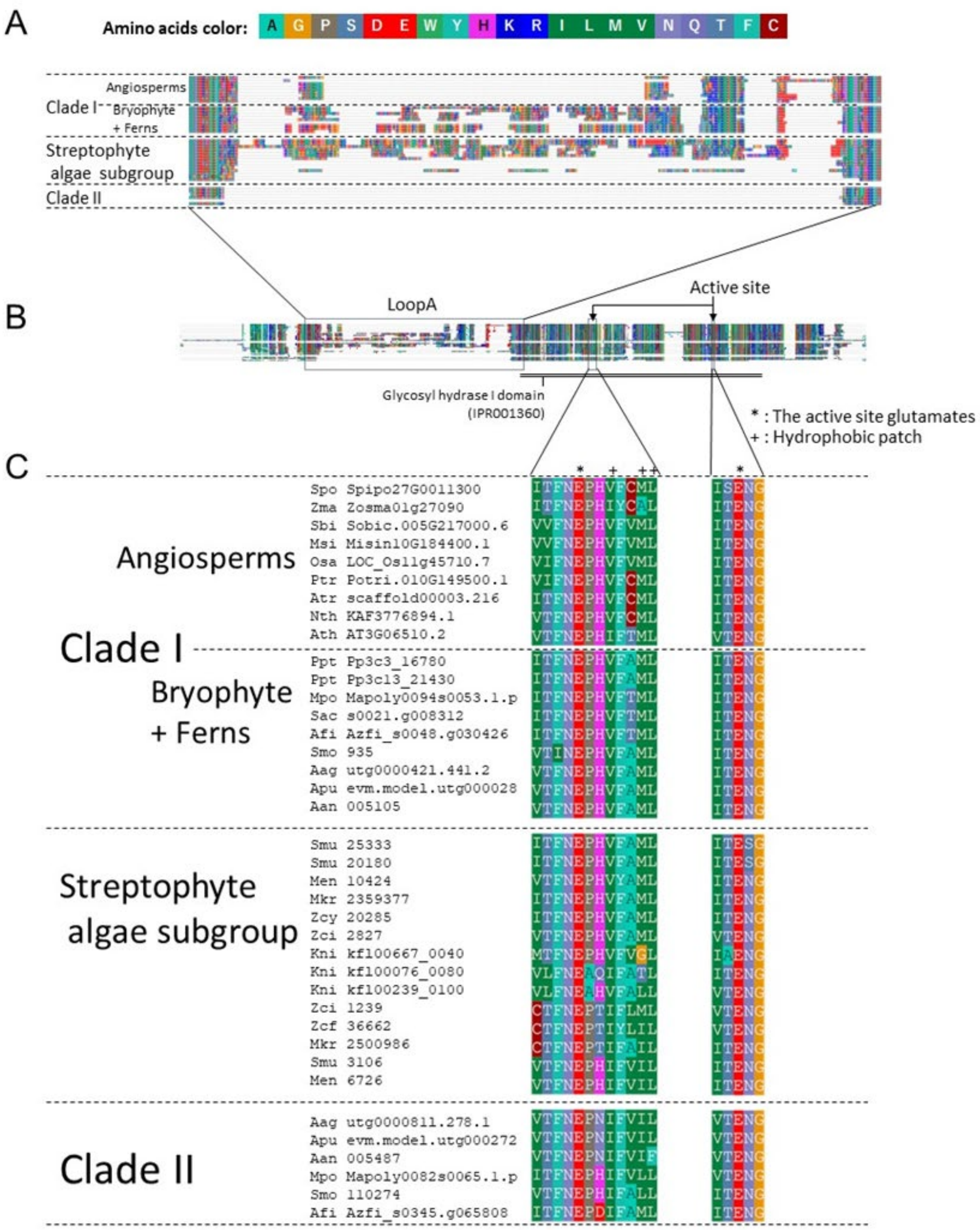

Figure S1. (A) Multiple sequence alignment of the Loop A region in 38 SFR2-like proteins. Amino acids are colored as indicated at the top. (B) Reconstruction of the multiple sequence alignment of the entire region, excluding Azfi_s0048.g030426, Aan 005105, Men 10424, Apu evm.model.utg000272, and Aan 005487, which predicted long insertions. Rectangles denote Loop A and the active site. The horizontal double line denotes the glycosyl hydrase I domain (IPR001360). (C) Multiple sequence alignment nearby the active-site glutamates and hydrophobic patch.

Table S1

Classification / Organism / Database	Abbreviation	BLAST site	Protein ID
Streptophyte algae			
Klebsormidium nitens	Kni	PhycoCosm	
Klenit1_1_GeneCatalog_proteins_20230514.aa1			kfl00667_0040
			kfl00076_0080
			kfl00239_0100
Mesotaenium endlicherianum	Men	PhycoCosm	
Mesen1_1_GeneCatalog_proteins_20230514.aa			10424
			6726
Mesotaenium kramstae Lemmermann	Mkr	PhycoCosm	
Meskra657_3_GeneCatalog_proteins_20220510.aa			2359377
			2500986
Zygnema cf. cylindricum	Zcy	PhycoCosm	
Zygcyl6981a_1_GeneCatalog_proteins_20230203.aa			20285
			36662
Zygnema circumcarinatum	Zci	PhycoCosm	
Zygcir1559_1_GeneCatalog_proteins_20230203.aa			2827
			1239
Spirogloea muscicola	Smu	PhycoCosm	
Spimu1_1_GeneCatalog_proteins_20230519.aa			20180
			25333
			3106
Bryophyte			
Anthocerotopsida			
Anthoceros agrestis	Aag	MarpolBase	
Anthoceros agrestis Oxford (v1.0, 2020)			utg0000811.278.1
			utg0000421.441.2
Anthoceros angustus	Aan	MarpolBase	
Anthoceros angustus (v1.0, 2020)			AANG005105
			AANG005487
Anthoceros punctatus	Apu	MarpolBase	
Anthoceros punctatus (v1.0, 2020)			utg0000281.432.1
			utg0002721.97.1
Hepatopsida			
Marchantia polymorpha	Mpo	MarpolBase	
Marchantia polymorpha 'primary' (v3.1, 2015)			Mapoly0094s0053.1.p
			Mapoly0082s0065.1.p
Bryopsida			
Physcomitrium patens	Ppa	MarpolBase	
Physcomitrium patens 'primary' (v3.3, 2018)			Pp3c3_16780
			Pp3c13_21430
Ferns			
Lycophytina			
Selaginella moellendorffii	Smo	MarpolBase	
Selaginella moellendorffii 'primary' (v1.0, 2014)			935
			110274
Polypodiopsida			
Azolla filiculoides	Afi	MarpolBase	
Azolla filiculoides (high_confidence) (v1.1, 2018)			Azfi_s0048.g030426
			Azfi_s0345.g065808
Salvinia cucullate	Scu	MarpolBase	
Salvinia cucullata (high_confidence) (v1.2, 2018)			s0021.g008312
Angiosperms			
Amborella trichopoda	Atr	MarpolBase	
Amborella trichopoda (v1.0, 2013)			scaffold00003.216

Nymphaea thermarum	Nth	MarpolBase	
Nymphaea thermarum (v1.0, 2020)			KAF3776894.1
Eudicots			
Arabidopsis thaliana	Ath	Phytozome	
Arabidopsis thaliana TAIR10			AT3G06510.2
Populus trichocarpa	Ptr	Phytozome	
Populus trichocarpa v4.1			Phpat.003G063100.1.p<br>Phpat.013G073100.1.p
Monocotes			
Zostera marina	Zma	Phytozome	
Zostera marina v3.1			Zosma01g27090
Miscanthus sinensis	Msi	Phytozome	
Miscanthus sinensis v7.1			Misin10G184400.1.p
Sorghum bicolor	Sbi	Phytozome	
Sorghum bicolor v5.1			Sobic.005G217000.6
Oryza sativa	Osa	Phytozome	
Oryza sativa v7.0			LOC_Os11g45710.7
Spirodela polyrhiza	Spo	Phytozome	
Spirodela polyrhiza v2			Spipo27G0011300

Table S2

Primer	sequence
MpGGGT1 Fwd	AAAAAGCAGGCTCAAAAATGGCGCCGTTTTTGTTGCTG
MpGGGT1 Rev	AGAAAGCTGGGTTATCGGCTGCTGCTGTAGCTCTCT
MpGGGT2 Fwd	AAAAAGCAGGCTCAAAAATGGCGATTTCCTTGAGGAAG
MpGGGT2 Rev	AGAAAGCTGGGTTATCAACTCCCCGGTCCTAAGTTC
SFR2 Fwd	GGGGACAAGTTTGTACAAAAAAGCAGGCTCAATGGAATTATTCGCATTGTTAATTAAGGTCG
SFR2 Rev	GGGGACCACTTTGTACAAGAAAGCTGGGTGGTCAAAGGGTGAGGCTAAAGCAGG
AtRbcs Fwd	AAAAAGCAGGCTCAAAAATGATGATAACTCGCGGTGG
AtRbcs Rev	AGAAAGCTGGGTTATCATGATACCCAATTGGAGCTG

Table S3

Primer	sequence
MpGGGT1 sgRNA Fwd 3rd exon 8	CTCGATTCCGTTGAGTGGCTCAAC
MpGGGT1 sgRNA Rev 3rd exon 8	AAACGTTGAGCCACTCAACGGAAT
MpGGGT2 sgRNA Fwd 1st exon	CTCGTGGAGACGGCTCCCGGAGAT
MpGGGT2 sgRNA Rev 1st exon	AAACATCTCCGGGAGCCGTCTCCA
